# When Holding in Prevents From Reaching Out: Emotion Suppression and Social Support-Seeking in Multicultural Groups

**DOI:** 10.3389/fpsyg.2019.02431

**Published:** 2019-10-25

**Authors:** Smaranda Boroş, Lore van Gorp, Michael Boiger

**Affiliations:** ^1^Area People and Organisation, Vlerick Business School, Ghent, Belgium; ^2^Department of Marketing, Innovation and Organisation, Faculty of Economics and Business Administration, Ghent University, Ghent, Belgium; ^3^Department of Sociology, Ghent University, Ghent, Belgium; ^4^Programme Group Social Psychology, Faculty of Social and Behavioural Sciences, University of Amsterdam, Amsterdam, Netherlands

**Keywords:** emotion suppression, individualism – collectivism, social support, similarity attraction, multicultural groups

## Abstract

Members of multicultural groups benefit from developing diverse social support networks. Engaging openly with people who have a different worldview (i.e., given by a different cultural background) broadens one’s cognitive horizons, facilitates one’s adaptation to new contexts, decreases stereotyping and discrimination and generally improves individual and group performance. However, if this social connection is hindered (either by limiting the number of people one reaches out to or in terms of preferring to connect to similar others), then the diversity advantage is lost – both for the individuals and for the groups. Through two case studies of professional groups with varying cultural diversity (moderate and superdiverse), we investigate the evolution of their members’ social support networks (i.e., to what extent and to whom they reach out for support) depending on (1) individuals’ habitual emotion suppression and (2) cultural orientation on the individualism-collectivism dimension. Results show that individualistic cultures suffer a double-whammy: when suppressing, their members seek less support (i.e., don’t reach out so much to ask for support) and tend to seek culturally similar others for it when they do. Suppressing collectivists are less affected in absolute levels of connectedness, but still prefer culturally similar others as sources of support. Our study offers an emotion-based view of why people stick together with similar others in diverse groups and how learning to better cope with emotions can make us more open-minded toward diversity in professional settings.

## Introduction

In a world where cultural diversity in professional settings is more often than not the norm, it is crucial to have in-depth understanding of the role emotional dynamics play in multicultural groups. However, research on emotional processes in multicultural groups is so far scant (Elfenbein and Shirako, 2006; Fischbach, 2009). This may be surprising, given the growing attention to both the topic of emotions in organizations (Ashkanasy et al., 2017) and that of cultural variation in emotions (Elfenbein and Ambady, 2002; Van Hemert et al., 2007; Mesquita et al., 2017) in the past two decades.

Multicultural groups challenge our most basic assumptions about the “rules of the game” of working together (Stahl et al., 2010).^1^ These challenges often come with intense emotions for members of multicultural groups, and group members may reach out to others in their group for support in handling and making sense of these emotions (Rimé, 2009).

Seeking social support in distressing situations offers a double-win. To the support seeker, it offers emotion support and help with emotion regulation; it also provides an opportunity to cognitively elaborate on experiences, which may help to better adapt to the situation. For the larger group to which the individual belongs, social support exchanges are the canvas that allow stronger and longer-lasting relationships to be built (Ibarra, 1993), newcomers to be socialized into organizational culture, organizational culture to be propagated and strengthened (Morrison, 2002), and groups to become more cohesive (Hogg, 1992).

It follows logically then that building a diverse social support network would be an optimal way both at individual and at group level to cope with emotions in multicultural groups, be they teams or larger social groups (such as communities). Diverse support networks makes adaptation to a new environment – be that professional (Ibarra, 1993) or even cultural (De Leersnyder et al., 2011) faster and swifter; it has a positive impact on work performance and career prospects (McDonald and Westphal, 2003), as well as general well-being (Cohen and Janicki-Deverts, 2012). However, reality shows this is rarely the case (Leung and Wang, 2015), and multicultural groups remain the terrain of separation and faultlines. Going beyond cognitive and social categorization analyses of this process, we focus instead on the emotional processes that impact the development of diverse social support networks. More precisely, we set out to explore how the individual emotion coping styles of members shape the emergence of their social support networks with dissimilar others in multicultural groups.

Research shows that notwithstanding the benefits of looking for support as an emotion regulation and meaning creation strategy, more basic, intrapersonal strategies are often the preferred, easier alternative. Such automatic strategies are primarily directed at modifying the outer expression of the emotion (i.e., emotion suppression), in order to respond appropriately to the situation (Gross, 2002). Emotion suppression consists of controlling or neutralizing emotional behavior (Gross and John, 2003; Matsumoto et al., 2008) and of actively inhibiting the observable expression of the emotional experience (Gross and Thompson, 2007). This is often a desirable and adaptive strategy in social interactions – for instance, when suppressing anger in front of a colleague or when wanting to hide anxiety before a big presentation. However, emotion suppression comes at both intrapersonal (Gross, 2002) and interpersonal costs, especially when it is a habitual regulation mechanism (i.e., habitual suppression). At interpersonal level, research has shown that *habitual suppression interferes with one’s engagement in social relationships*, both by disrupting the dynamics of social interactions and existing relationships (Butler et al., 2003; Srivastava et al., 2009), and by preventing habitual suppressors from forming social connections in the first place (English et al., 2012; Tackman and Srivastava, 2016). In other words, habitual suppression appears to hinder the establishment of social support networks thereby limiting interpersonal regulatory strategies such as support seeking.

However, the exact *consequences of habitual suppression appear to vary across cultural contexts* (Butler et al., 2007). For example, in an European American context, suppression is associated with higher levels of depression and lower levels of life satisfaction. However, for Hong Kong Chinese, for whom suppression is instrumental to adjusting to others, suppression is not associated with depression or less life satisfaction (Soto et al., 2011). These cultural differences may be explained by the different role that self-expression plays in these contexts (Tsai and Lau, 2013): emotion suppression violates the individualistic norm for self-expression, while it is generally in line with collectivistic norms of self-adjustment.

This evidence focuses on the impact of emotion suppression on the individuals themselves (e.g., well-being, job satisfaction, levels of depression). The question remains *how the differential preferences for suppression depending on culture of origin will impact people’s interpersonal behavior in multicultural groups*. We know that a basic condition for reaping the benefits of diversity in groups is engaging openly with people who have a different worldview – e.g., with a different cultural background (Stahl et al., 2010). If this social connection is limited by emotion suppression (either by *limiting the number* of people one reaches out to or in terms of *preferring to connect to similar others*), then the diversity advantage is lost (Cox and Blake, 1991; Ely and Thomas, 2001; Ashkanasy et al., 2002).

To address this gap, the present paper attempts to contribute to the advancement of knowledge in the field in two ways: First, we replicate previous research of how habitual suppression impacts the search for social support in professional groups in a longitudinal network study design. Second, we explore the role of culture in this relation. Based on previous findings about the most relevant cultural dimensions with differential impact on the consequences of habitual suppression (Butler et al., 2007; Matsumoto et al., 2008; Soto et al., 2011), we will look specifically at differences in individualism – collectivism in one’s culture of origin. Moreover, we treat culture both as a value lens (i.e., how cultural values from the primary socialization influence the impact of habitual suppression on seeking social support in professional groups) as well as an indicator of group diversity: i.e., we will look at culturally homogenous, moderately diverse (i.e., there is a dominant culture) and superdiverse^2^ (there is no dominant culture) groups. To do so, this study presents four case studies. For each case study the members of a particular group were asked to fill out a survey at three or four moments in time, resulting in longitudinal network data which allows us to take into account structural characteristics of network dynamics and to look at the dynamic process of selecting support providers. By adopting a network methodology, we also answer the call for emotion research in organizations to go beyond experiments (Webb et al., 2012) and cross-sectional surveys (Hu et al., 2014) and use novel methods that allow for more comprehensive reflections on the context (social, organizational, cultural) in which these emotional dynamics play in professional settings (Ashkanasy et al., 2017).

In sum, our study aims to answer the following question: How does habitual suppression impact the evolution of people’s social support networks in culturally diverse groups? To this aim, we look at (1) the influence of habitual suppression on looking for new support providers, (2) the role someone’s cultural background plays in this relationship, as well as (3) at patterns of preferential connections (to whom one reaches for support), thereby testing propositions set forth by cultural homophily research (McPherson et al., 2001).

### Suppression and Social Engagement

Emotion suppression, while quick to be activated and easily employed, comes with an array of costs – especially to those for whom self-expression is a core value (Markus and Kitayama, 1991). In the following section, we build on the main findings regarding these costs and explain how intrapersonal costs (i.e., cognitive and affective) impact the social functioning and eventually the adaptability and integration of the habitual suppressor (so the intrapersonal costs). To start, we review the main effects of suppression that previous research has highlighted. The next sections refine these findings by adding the cultural dimension, first by discussing the impact of cultural dimensions on the main effect of suppression, and afterward by looking at variation in group members’ culture of origin as a proxy for cultural diversity in groups.

Research (mainly conducted in individualistic cultures – e.g., North American) has overwhelmingly proven thus far that habitual emotion suppression interferes with one’s engagement in social relationships: Previous studies demonstrated that suppression not only disrupts the dynamics of social interactions and existing relationships (Butler et al., 2003; Srivastava et al., 2009), but also prevents habitual suppressors from forming social connections in the first place (English et al., 2012; Tackman and Srivastava, 2016).

Emotion suppression impairs social functioning via both affective and cognitive mechanisms. Because suppression decreases both negative and positive emotion-expressive behavior, it thereby ends up masking important social signals that would otherwise be available to social interaction partners. This affects both the partner as well as the suppressor, who comes to feel inauthentic in interactions (affective mechanism) (English and John, 2013). The self-monitoring and self-regulation process is taxing on an individual’s cognitive resources, making thereby the suppressor less attentive to and less responsive to the partner’s emotional cues –i.e., the cognitive mechanism (Butler et al., 2003; Richards et al., 2003). This in turn makes the partner feel detached or unappreciated (Impett et al., 2012). Both mechanisms point to losses in the richness of the interaction with a suppressor. In time, this leads habitual suppressors to withdraw from the kinds of reciprocal disclosure that would otherwise promote intimacy and closeness (English and John, 2013). Therefore, individuals who habitually suppress are less likely to share either their negative or their positive emotions with others, which in time leads to having poorer social support and making lesser use of social support coping (Gross, 2002). In a longitudinal study on college adaptation, Srivastava et al. (2009) attested that individual differences in suppression predicted difficulties forming close relationships and getting social support in the new college environment (Srivastava et al., 2009). At the end of college, habitual suppressors ended up having less social support, less satisfying social lives, and experienced trouble getting close to others (Tackman and Srivastava, 2016).

### Quantifiers of Emotion Suppression Costs: A Cultural Value Lens Analysis

Research conducted with participants from individualistic countries shows that people feel inauthentic and are seen as inauthentic when they do not express themselves (English and John, 2013). However, acts of self-expression enhance perceptions and feelings of authenticity only when they are congruent with the culturally prevalent self-expression norms (Kokkoris and Kühnen, 2014). These norms differ radically between individualistic cultures, which promote an independent construction of the self, and collectivistic cultures, which promote an interdependent construction of the self (Matsumoto, 1990; Markus and Kitayama, 1991).

Values in individualistic cultures emphasize self-affirmation, the pursuit of individual goals and open emotional expression (Frijda and Sundararajan, 2007). They promote a strong shared belief in the independence of the self from others and therefore the major cultural quest is to discover, actualize, and confirm these internal attributes of the self (i.e., the independent view of self, cf Markus and Kitayama, 1991). Since the individualistic values of independence and self-assertion encourage open self-expression, it follows that emotion suppression has primarily a self-protective function in these cultures: i.e., people suppress the expression of their emotions as an act of withdrawal from a social threat (Matsumoto, 1990; Markus and Kitayama, 1991; Tsai and Levenson, 1997; Oyserman et al., 2002; Butler et al., 2007). Therefore, suppression is associated with avoidant attachment, which involves a lack of trust in others and a tendency toward social withdrawal (Gross and John, 2003), less social closeness and support (John and Gross, 2004), reduced rapport, and inhibited relationship formation (Butler et al., 2003).

However, the situation looks different in collectivistic cultures, where cultural values emphasize social connection, in-group harmony, individual restraint and suppression of socially inappropriate emotions (Hu et al., 2014). In these cultures, which hold relational harmony as a primary value, individuals are encouraged to take their proper place in the community. Expansive behavior and emotional expression are seen as “taking too much space,” and thus condemned (Mesquita and Walker, 2003). Thus, suppression is not only more frequent than in individualistic cultures (Butler et al., 2007), but it is in fact in line with the fundamental values of the culture.

Since collectivistic cultures promote primarily connectedness or interdependence among those within an ingroup, the major cultural quest is to adjust to relationships and become a proper member of the group (Morling et al., 2002; Boiger et al., 2012). To do this, one must constrain, tame, or otherwise condition internal desires or wishes that may in any way hinder interpersonal harmony and unity (i.e., the interdependent view of self, cf. Markus and Kitayama, 1991). Therefore, the collectivistic values of interdependence and relationship harmony will encourage suppression equally often for prosocial goals (e.g., hiding glee when winning, suppressing anger to preserve a relation) and during positive social interactions, rather than constraining it only to self-protective purposes. When people from collectivistic cultures engage in suppression, it is most often out of concern about hurting someone else, and trying to preserve relationships (Butler et al., 2007). This “other-protective” goal has been shown to even revert the consequences of suppression, by being associated with personal well-being and relationship quality in close relationship (Le and Impett, 2013) rather than decreased feelings of rapport and affiliation, and increased negative feelings about the interaction (Butler et al., 2003).

Because of the ensuing differences in the characteristics and interpretations of suppression, their consequences have been proven radically different for individualistic and collectivistic cultures (see Hu et al., 2014, for a meta-analysis). For example, suppression was correlated negatively with mental health (e.g., life satisfaction, and positive affect) and positively with distress (e.g., depression, anxiety, and overall negative affect) in studies on samples from individualistic cultures, but not in samples of participants from collectivistic ones. In other words, evidence indicates that culture (and in particular the individualism-collectivism dimension) is one of the most relevant moderators of the impact of emotion suppression on individuals’ well-being and social functioning. Along these lines, there is a common belief that gender norms generally differentiate between men and women’s socially condoned expression of emotions. When it comes to suppressing these expressions, significant effects for habitual suppression were found across gender in Western, individualistic samples (Gross and John, 2003). However, in cross-cultural meta-analyses (Matsumoto et al., 2008), while gender differences persisted, they were not large enough to be reflected in country-level data (within-country differences on gender were smaller than between-country differences), indicating that culture is a more significant moderator of emotion suppression than gender or age (despite both these variables being relevant in individual differences of emotion suppression). Considering extant evidence on the importance of social support for one’s well-being and organizational functioning (Kahn et al., 2006; Hayton et al., 2012), as well as the role suppression plays in limiting access to social support networks (Butler et al., 2003; Srivastava et al., 2009), it is imaginable that the less detrimental effects on well-being in collectivist cultures are due to habitual suppressors withdrawing less from support networks. Based on the different dynamics that play in individualistic and collectivistic cultures, we predict that:

Hypothesis 1: *In culturally diverse groups, the level of Individualism/Collectivism of one’s country of origin moderates the relation between emotion suppression and support seeking in such a way that people from more individualistic cultures who suppress their emotions will ask less others for support, while the impact of emotion suppression on support seeking is smaller for people from more collectivistic cultures.*

### Cultural Homophily Effects of Habitual Suppressors

The intrapersonal consequences of emotion suppression impact not only the extent to which suppressors reach out for support, but also who they reach out to: Habitual suppressors, who avoid support seeking in most situations, may additionally hamper their chances by reaching out to similar others when they seek support at all. Previous research evidenced repeatedly that a diverse network to tap into for personal support improves people’s adaptation to a new environment – be that professional (Ibarra, 1993) or even cultural (De Leersnyder et al., 2011) –, work performance and career prospects (McDonald and Westphal, 2003), as well as general well-being (Cohen and Janicki-Deverts, 2012). When suppressing, however, people may prefer to reach out to those who hold similar worldviews, thereby avoiding to engage in social relations that would have proved more helpful and adaptive in the longer run (Rimé, 2009). So why is that the case? Again, a cognitive and an affective path of suppression costs explain this phenomenon.

Because of the extra strain imposed on the cognitive system by suppression, limited resources are available to process further information (Gross, 2002). Cognitive functioning impaired by suppression means that in more complex processes, such as how we make choices and social judgments, we rely mainly on automatic heuristics (Hofmann et al., 2007). Evidence from similarity-attraction paradigm studies shows that increased similarity with a target (even a total stranger) – with respect to attitudes, personality traits, or even demographic attributes – is associated with increased attraction to the target (Byrne, 1997). This attraction is automatic, fast, based on one or few salient cues to similarity, and happens through an affective processing path. This automatic processing leads then to reaching out to people holding similar cultural values and attitudes for social support instead of considering who the best person would be to offer that support – i.e., using the cognitive path, which requires deliberation and thought. This automatic processing is one of the possible explanations behind why immigrants for instance choose to reach out to other co-nationals, instead of host country nationals, thereby making their cultural adaptation in a new country more difficult (De Leersnyder et al., 2011).

There is also an affective mechanism which explains the preference for homophily in support networks following emotion suppression. According to Rimé (2009), socio-affective sharing “contributes to the fulfillment of the socio-affective needs of the narrator by providing him or her with responses that offer help, support, comfort, consolation, legitimization, attention, bonding, and empathy” (Rimé, 2009, p. 47). Sharing your problems and relying on support from a person with different attitudes and world-views can thus help the advice seeker broaden their understanding of a situation, and develop new operating schemata and behavioral alternatives. This comes, however, at a cost: the person does not receive immediate alleviation in the interaction: on the contrary, rather than being validated in their emotional experience, they would be challenged further (Rimé, 2009). A lack of emotional and cognitive resources due to suppression prevents suppressors from entering such challenging interactions, making it more likely they will turn to the sources who will offer them comfort and validation instead – so others with similar attitudes and values.

In light of these mechanisms, we hypothesize that:

Hypothesis 2: *In culturally diverse groups, habitual suppressors have a stronger preference to develop a support network with culturally similar others than non-habitual suppressors.*

## MATERIALS AND METHODS

### Procedure

To investigate the influence of emotion suppression on the development of a support network and the role of individualistic-collectivistic primary socialization in this relation, data were gathered with two different groups: one moderately diverse (i.e., one dominant individualistic culture and a minority of different other nationalities) and one superdiverse group (i.e., no dominant culture/nationality). In order to be able to assess the dynamic process of network development, longitudinal data were collected for each case. The group of MBA participants filled-out a questionnaire at three moments in time. The second, consisting of marketing students (post-masters professional education), filled out a survey at four points in time.

### Participants

The two groups were newly formed groups and were both enrolled in a full-time (MBA or Marketing Track) program in a Belgian Business School. The participants in these programs (i.e., young professionals who take a 1 year sabbatical to undergo these executive education programs) had daily contact during a whole academic year. The MBA students filled out a questionnaire at the beginning of the academic year, a second one two and a half months later and the last one at the end of their plenary sessions (i.e., 6 months after the first one). For the marketing students, one additional measurement was scheduled, i.e., in between the first and second measurement point of the MBA sample. As presented in Table 1, the group of marketing students consisted of 58 students, of whom 37.9% were male and the average age was 23.1. Regarding country of birth the marketing group was *moderately diverse* with one dominant culture, as 43 of them (74.1%) were born in Belgium and the other 15 students were born in 12 different countries. The group of MBA students comprised 65 participants, of whom 63.1% were male with an average age of 29.2. This group was also the more diverse group in terms of country of birth, only 8 MBA-students (12.3%) having been born in Belgium and the 57 other MBA-students born in 26 different countries (i.e., a *superdiverse* group).

**TABLE 1:** Sample characteristics.

	**Marketing students**	**MBA students**
		
	***M*(SD) or %**	***M* (SD) or %**
Age	23.1 (1.2)	29.2 (2.8)
Membership (in years) group t1	<2 weeks	<2 weeks
Gender – male	37.9%	63.1%
**Country of birth**		
Belgian	74.1%	12.3%
The Netherlands	1.7%	–
Germany	3.4%	3.1%
Spain	1.7%	–
India	3.4%	13.8%
Russia	3.4%	7.7%
United States of America; Colombia	1.7%	6.2%
China	1.7%	3.1%
Australia; France; Hungary; Slovakia	1.7%	–
Romania		7.7%
Kazakhstan; Taiwan; Greece; South Africa; Georgia; Vietnam; Azerbaijan		3.1%
Hungary; Thailand; Austria; Peru; Brazil; Cyprus; Ukraine; New-Zealand; Malaysia; Moldova; Indonesia; Chile		1.5%
	*N* = 58	*N* = 65

*For economy of space, countries are presented together in the same cell when they share the same representation value in the sample.*

### Measures

#### Habitual Emotion Suppression

At the second data-collection point, the Emotional Regulation Questionnaire (Gross and John, 2003) was included in the surveys. Four out of these ten items are used to measure emotion suppression. All four suppression items were measured on a 7-point Likert scale ranging from “Strongly disagree” (= 1) to “Strongly agree” (= 7). A sample item is “I keep my emotions to myself.” As presented in Table 2, the Cronbach’s alpha for this scale is sufficiently high in both samples: 0.81 and 0.82 respectively.

**TABLE 2:** Descriptive statistics.

	**Marketing students**			**MBA students**		
		
	**$\bar{x}$**	**SD**	**α**	**$\bar{x}$**	**SD**	**α**
Emotion suppression	3.31	1.24	0.81	3.72	1.41	0.82
Collectivism-individualism	/			3.38	1.81	
Response rate						
t 1		98.3%			90.8%	
t 2		100%			90.8%	
t 3		89.7%			81.5%	
t 4	93.1%					

**Social support network:**	**Nr support ties (δ)**	**Density δ/(n^∗^n-1)**	**Nr support ties (δ)**	**Density δ/(n^∗^n-1)**

t 1	91	0.03	106	0.03
t 2	222	0.07	120	0.03
t 3	193	0.07	153	0.05
t 4	271	0.09		

	**Hamming Distance**	**Jaccard**	**Hamming Distance**	**Jaccard**

t 1-t 2	208	0.19	153	0.16
t 2-t 3	191	0.33	132	0.32
t 3-t 4	189	*0.39*		
	*N* = 58		*N* = 65	

#### Cultural Similarity I (Belgian – Non-Belgian)

In the marketing group there is a *moderate diversity* regarding country of birth; in this group, 74.1% of participants were born in Belgium. With one dominant cultural group, the most salient distinction between members was assumed to be non-Belgian vs. Belgian. Consequently, we operationalized cultural similarity in this group as a dichotomized variable, namely non-Belgians (= 0) vs. Belgians (= 1) and respondents from the same group are considered to be culturally similar.

#### Cultural Similarity II

The MBA-group is a *superdiverse* group regarding country of birth as there is no one dominant country of birth and the 65 students are born in 27 different countries. Cultural similarity in this group is based on a metric variable, namely Hofstede’s country values on the dimension collectivism-individualism (Hofstede et al., 2010), and respondents are considered to be more cultural similar the more comparable their collectivism-individualism score. We used this proxy for the value system in which someone got his/her primary socialization following similar research conducted by Tröster et al. (2014). The initial Hofstede’ country values ranged between 13 and 91 and were rescaled by dividing them by 13 in order to have the same range as the other measures used (i.e., 1–7). A low score on this variable indicates that someone is born in a more collectivistic culture, and a high score points to a more individualistic culture.

#### Social Support

In line with research of De Lange et al. (2004) social support was measured by giving respondents a roster with the names of all group members (i.e., recognition method) and asking them to put a check mark next to “*each person you repeatedly consulted for help and support on personal-related problems, such as for example relational problems, death of a beloved one, lack of motivation, problematic relation with another student*,…*.”* Respondents were asked to make a binary judgment for each of their fellow group members. We chose binary judgments because they are less difficult for respondents (Marsden, 2005) and thus help avoid respondent fatigue or drop-out for this network panel design. Social support was measured in this way during each data-collection point. In Table 2, the number of reported support ties are presented for each group and each data-collection moment, as well as the density of the social support network, that is, the number of actual ties divided through the number of possible ties.

### Descriptive Statistics and Analytic Strategy

Surveys were distributed on paper and collected in blank, sealed envelopes. As participants shared personal info on the survey itself (to allow for longitudinal data integration), we also guaranteed participants anonymity of their results – i.e., sharing them only as a group pattern. Participation was voluntary and response rates are relatively high, varying between 74.1 and 100% as shown in Table 2. As a response rate of 70% is needed to perform longitudinal network analyses (Kossinets, 2006), the data of this study meet this criterion.

To test our hypotheses, we used stochastic actor-based models. The models capture network dynamics over time and are most appropriate for complete network data. Social support is measured in this study by collecting data on whole networks, as a roster of the names of all group members is used in each group. Data on this social support measurement resulted for each group and for each wave in a binary adjacency matrix (i.e., a square matrix that represents which ties are present and which not between all possible pairs of the network) of size N^∗^N, so for instance for the MBA group the social support variable results for each wave in a table with 65 rows and 65 columns. If someone (i.e., ego *i)* indicated to rely on someone else (i.e., alter *j*) for social support, the cell *x*_*ij*_ got code 1. If there was no relationship (i.e., no tie from *i* to *j)* the cell was coded 0. Given the specific structure of this measurement in combination with the different data-collection moments, stochastic actor-based models for network dynamics are most appropriate and analyses are executed according to the procedures described in Ripley et al. (2011) and Snijders et al. (2010) with RSiena (version 1.1-232).

The density indicators of the social support network in Table 2 show both intense support-seeking activity in both groups, and that the number of support ties increases over time in both samples. When applying stochastic actor-based models for network dynamics, networks need to change over time but they also need to maintain a certain stability (Ripley et al., 2011). The Hamming distance coefficients (i.e., the indicators for the amount of tie changes between two periods) were above the 0 threshold, and the Jaccard coefficients (i.e., the indicator for network stability, which takes into account both the number of changing ties as well as the number of stable ties) were above 0.1, indicating that there is both enough stability and change to consider the data as an evolving network and to apply stochastic actor-based models for network dynamics. The convergence of the estimation algorithm was excellent for all models presented (all t-ratios < 0.1).^3^

Typical for stochastic actor-based models is to include some basic structural effects that are often at play in a social network. As such, some structural endogenous network effects are included as control variables in this study, namely an effect for outdegree, reciprocity, transitive triplets effect and 3-cylces. Table 3 shows a visual representation of these effects as well as an explanation. Moreover, for traditional variables different effects can be included. For instance, gender can be included as a characteristic of the person who is asking someone else for social support, that is someone who is sending a tie. In this case, the effect is called an “ego-effect.” However, gender can also be a characteristic of the person who is being asked for social support, that is the person who receives a tie from someone else. In these cases, the gender-effect is called an “alter-effect.” Finally, when combining both the information of ego and alter a third effect can be calculated for the same variable, namely a “similarity-effect” which represents the similarity or difference between alter and ego. Both for age and gender all three effects are included as controls in the analyses.

**TABLE 3:** Explanation and visualization of network effects.

**Effect**	**Explanation**	**Visualization**	
Outdegree	Tendency to create new support ties to arbitrary others. Outdegree is the effect that indicates how many new ties there are formed to randomly another actor in the network)	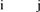	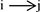
Reciprocity	Tendency to ask for support from someone that asked you already for support. Reciprocity is the indicator showing that if someone asks you for support you are more likely to ask that person for support in the future than a random other person for the group	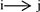	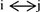
Transitive triplets	Tendency of i to ask for support from the support provider of a current support provider. Transitive triplets effect refers to the phenomenon that if A asks B for support and B ask C for support than it is more likely in the future that A will ask C for support than a random other in the network	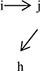	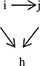
3-cycle effect	Tendency that is asked for support by the support provider of his own support provider. 3-cycle-effects refer to the effect that if A asks B for support and B asks C for support C is more likely to ask A for support than a random other	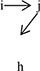	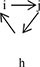

To test Hypothesis 1 – “In culturally diverse groups, the level of Individualism/Collectivism of one’s country of origin moderates the relation between emotion suppression and support seeking in such a way that people from more individualistic cultures who suppress their emotions will ask less others for support, while the impact of emotion suppression on support seeking is smaller for people from more collectivistic cultures.” We calculated the interaction term between the “ego-effect” of emotion suppression and the “ego-effect” of the individualism/collectivism score. This hypothesis can only be tested in groups that are highly culturally diverse and as such we tested it in the superdiverse (i.e., MBA) sample only.

We tested Hypothesis 2 – “In culturally diverse groups, habitual suppressors have a stronger preference to develop a support network with culturally similar others.” By calculating an interaction term between the “ego-effect” of emotion suppression and the “similarity-effect” of the individualism/collectivism score. We took into account that it may be possible for this effect to be limited to the specific case where there is no culturally dominant group: in this case it may be particularly tempting to be associated with culturally similar others, and little pressure to engage with dissimilar others. However, we wanted to test if this was also true in a setting where one could assume more pressure to associated with the culturally dominant group. Therefore, Hypothesis 2 is tested in both the superdiverse (MBA) and moderately diverse (Marketing) groups.

## Results

We proposed that the negative effect of suppression on social support network expansion would vary depending on the cultural values regarding individualism/collectivism in which people got their primary socialization. To investigate if the impact of suppression is different for people coming from more collectivistic vs. more individualistic cultures when they are interacting together in a multicultural group we focus on the superdiverse case, that is the group of MBA students. Results (presented in Table 4) suggest that the influence of suppression on the likelihood of looking for additional support providers is influenced by someone’s cultural background. More precisely, results suggest that especially for people from more individualistic cultures being a habitual suppressor prevents them to extend their social support network (*b_interaction_* = −0.07; *p* < 0.05). Therefore, results support Hypothesis 1.

**TABLE 4:** Dynamics of social support networks in culturally super-diverse and moderately diverse groups – unstandardized coefficients (standard errors).

	**MBA student**	**MBA students**	**MBA students**	**Marketing students**	**Marketing students**
					
	**Model1.I**	**Model1.II**	**Model1.III**	**Model2.I**	**Model2.II**
					
	**Est. (s.e.)**	**Est. (s.e.)**	**Est. (s.e.)**	**Est. (s.e.)**	**Est. (s.e.)**
Rate period 1 (t1-t2)	8.02 (1.31)	8.16 (1.27)	8.01 (1.26)	15.25 (3.20)	15.19 (2.86)
Rate period 2 (t2-t3)	6.01 (0.82)	6.18 (0.82)	6.04 (0.81)	6.94 (0.72)	6.98 (0.74)
Rate period 3 (t3-t4)				7.38 (0.79)	7.44 (0.78)
	(t1–t2–t3)	(t1–t2–t3)	(t1–t2–t3)	(t1–t2–t3–t4)	(t1–t2–t3–t4)
**ENDOGENOUS NETWORK EFFECTS**					
Outdegree (density)	−2.32(0.09)***	−2.34(0.09)***	−2.35(0.08)***	−2.19(0.08)***	−2.20(0.08)***
Reciprocity	1.74(0.18)***	1.74(0.18)***	1.73(0.18)***	1.80(0.13)***	1.79(0.13)***
Transitive triplets	0.54(0.11)***	0.52(0.11)***	0.53(0.12)***	0.52(0.05)***	0.52(0.05)***
3-cycles	−0.09(0.23)	−0.10(0.22)	−0.08(0.24)	−0.37(0.10)***	−0.36(0.11)***
**EXOGENEOUS NETWORK EFFECTS**					
Suppression ego	0.02 (0.04)	−0.01(0.05)	−0.04(0.05)	−0.06(0.03)*	−0.14(0.05)*
Collectivism-individualism ego	−0.01(0.04)	−0.04(0.04)	−0.02(0.04)		
Collectivism-individualism alter	0.01 (0.04)	0.02 (0.04)	0.01 (0.04)		
Collectivism-individualism sim	1.25(0.26)***	1.30(0.27)***	1.33(0.26)***		
Suppr. ego^∗^Collectivism-indiv. Ego		−0.07(0.03)*			
Suppr. ego^∗^Collectivism-indiv. sim			0.44(0.20)*		
Belgian - non-Belgian ego				−0.17(0.11)	−0.19(0.11)^+^
Belgian - non-Belgian alter				0.09 (0.10)	−0.09(0.09)
Belgian - non-Belgian same				0.19(0.09)*	0.20(0.09)*
Suppr. ego^∗^Belgian - non B. same					0.11(0.06)^+^
**CONTROL COVARIATES**					
Gender ego (male = ref. cat.)	0.14 (0.15)	0.06 (0.14)	0.15 (0.14)	−0.23(0.09)*	−0.25(0.09)**
Gender alter	−0.00(0.12)	−0.01(0.12)	−0.01(0.12)	0.03 (0.08)	0.04 (0.08)
Gender similarity	0.12 (0.11)	0.12 (0.11)	0.10 (0.11)	0.25(0.07)***	0.25(0.07)***
Age ego	−0.04(0.02)*	−0.04(0.02)*	−0.04(0.02)*	−0.03(0.04)	−0.03(0.04)
Age alter	−0.01(0.02)	−0.01(0.02)	−0.01(0.02)	−0.04(0.04)	0.04 (0.04)
Age similarity	0.35 (0.33)	0.32 (0.33)	0.36 (0.34)	0.11 (0.18)	0.14 (0.18)
	65			58	

*^+^p < 0.1; ^∗^p < 0.05; ^∗∗^p < 0.01; ^∗∗∗^p < 0.001. Example of interpretation: Positive significant similarity effect of collectivism-individualism score: People are more likely to look for additional support providers over time (t1–t2–t3) that are more similar to them regarding their cultural background on individualism-collectivism than randomly new support providers.*

To further zoom in on the influence of suppression on the development of social support networks in culturally diverse groups this study investigates in both a culturally superdiverse case as well as in a culturally moderately diverse case if habitual suppression influences someone’s preference to ask for support from culturally similar others. Results of the culturally superdiverse case show that, in general, groups members have a preference to extend their support network with culturally similar others (*b* = 1.25; *p* < 0.001). Moreover, results also suggest that this homophily preference is especially present the more people suppress their feelings (*b_interaction_* = 0.44; *p* < 0.05). Similar effects are found in the culturally moderate diverse case, that is the marketing students. Results of this case also show that in general people from the cultural dominant group prefer to extent their social support network with others from the cultural dominant group while groups members from the cultural minority groups prefer others from a minority group (*b* = 0.19; *p* < 0.05). In addition, results also suggest that, on a marginally significant level, the more someone suppresses his/her feelings the stronger their preference to extend their support network with others from the same cultural group (majority group vs. minority group) (*b*_*interaction*_ = 0.11; *p* < 0.10). Therefore, Hypothesis 2 is supported by the data.

## Discussion

Through two case studies of professional groups with different degrees of cultural diversity, we set out to investigate the evolution of social support networks depending on group members’ habitual emotion suppression and cultural orientation, as indicated by their culture of primary socialization’s individualistic or collectivistic tendencies. In line with previous studies conducted in individualistic settings (English et al., 2012; Tackman and Srivastava, 2016), our data confirmed that individualistic people who habitually suppress tend to expand less their support networks over time, whereas habitual suppressors coming from more collectivistic countries are less impacted by the negative social consequences of suppression (H1).

What holds true irrespective of a person’s culture of origin is a preference for habitual suppressors to seek out culturally similar others for social support (H2). In moderately diverse groups, this plays out as a simple “us vs. them” dichotomy (i.e., “Belgians vs. internationals,” in our sample). In superdiverse groups, the cultural homophily effect becomes more nuanced: suppressors from individualistic cultures reach out to others coming from individualistic cultures (irrespective if it is the same country as theirs or not), whereas suppressors from collectivistic cultures will reach out to other collectivists. Given that one’s culture’s degree of individualism and collectivism is not a visible personal attribute (like gender or age), what could explains this effect?

We link this finding with previous research on how a culture’s degree of individualism-collectivism shapes the view of self of its members, and how this in turn dictates desirable behaviors. Fundamentally, the source of self-esteem for collectivists is generically based on “getting along,” while for individualists it is based on “getting ahead” (Triandis, 2001). This means that collectivists’ *social interactions* are characterized by attentiveness and responsiveness to others, by continually adjusting and accommodating to these others in many aspects of behavior whereas for individualists, a primary need is to express one’s own thoughts, feelings, and actions to others rather than be at the receiving end of the interaction (Markus and Kitayama, 1991). Consequently, during *teamwork* or in relation to tasks in general, the former would likely focus more on group harmony while the latter on individual achievements and performance. Furthermore, for collectivists *conformity* to relevant in-group others can be a highly valued end state instead of a sign of weakness, as an individualist would see it (Markus and Kitayama, 1991). Finally, in *conflict* situations collectivists are primarily concerned with maintaining relationships with others, whereas individualists are primarily concerned with achieving justice (Triandis and Suh, 2002).

Observing these differences in the others’ behaviors in professional settings can easily lead to stereotyping of the “Other”: for instance, collectivists can see individualists as cold, too ambitious at the cost of relationships, rude and unfeeling in conflict management. Alternatively, individualists can see collectivists as not ambitious/driven enough, not having their own mind/opinion, and possibly unreliable and hypocritical as they don’t express their position transparently (Clausen, 2010). These stereotypes bring with them a level of discomfort in reaching out to someone you see as behaving in ways that your own cultural formatting deems as undesirable (Stahl et al., 2010), and this holds especially true for habitual suppressors, who will not want their worldviews challenged (Rimé, 2009). This may explain the cultural homophily effect we observed in our diverse groups. Qualitative research investigating the unfolding relation between suppression and stereotyping (how it happens, what are conditions that mitigate it), the emotional cycle triggered by it and how it plays out in subsequent interpersonal interactions would be valuable in exploring this further and in adding to the understanding of conflicts in diverse groups.

What are the *possible implications of cultural homophily* preferences in building social support networks for the *individuals* involved and for the *groups* they operate in? In his model of social sharing of emotions, Rimé (2009) distinguishes between cognitive sharing (i.e., listeners adopt responses that prompt cognitive work) and socio-affective sharing (listeners adopt socio-affective responses, which reassure the individual and offer emotional support but do not challenge the emoter’s existing schemata). Seeking similar others to share emotional episodes with, prompts more socio-affective sharing, as these listeners share a similar view of the world with the emoter. However, such a response – though reassuring and validating the experience of the emoter as well as buffering the emotional episode – would not change the initial appraisal of the event, nor would it close the gap in representations and schemata. Overall, “nothing would have contributed constructively to the search for meaning elicited by the episode” (Rimé, 2009, p. 79). Rimé predicts that such an interaction will fail to provide emotional recovery and the emotional impact of the episode that elicited the exchange could persist and reactivate the destabilization. This mechanism can be a possible explanation why migrants who seek social and emotional support preferentially from others from the same home culture have a more difficult time adapting emotionally and culturally to a new context (De Leersnyder et al., 2011).

Therefore, the observed pattern of suppressors to rely preferentially on culturally similar others to get social support, while offering immediate relief and a reduction of loneliness (Rimé, 2009), also risks in the longer run to *strengthen the shared beliefs with culturally similar others* in the network. In a moderately diverse group, this could lead to *difficulty in adapting* to the new culture by newcomers and minorities, as well as a reluctance of the majority to integrate them and accommodate different worldviews (Cooper et al., 2007).

In other words, culturally homophilous social support networks seem to have a double bind: offering emotional relief and support to individuals, while at the same time creating and perpetuating “*us vs. them*” construction, the emergence of *subgroups*, and polarization of opinions in groups (Haslam et al., 2006). These subgroups are all the more stronger since they are formed not just around task content, but also emotion ties, and inform ways of working together and informal connections between group members (McPherson et al., 2001). This finding is in line with previous research on both conflict and effectiveness in diverse groups: On the one hand, highly diverse groups with less dense networks see themselves as less able to solve the task at hand (i.e., lower team potency perceptions) (Tröster et al., 2014) On the other, highly diverse groups on the individualism dimension use less collaborating conflict resolution strategies to solve their disagreements (Boroş et al., 2010), thereby leading to more conflict escalation in time.

The structural effects we have evidenced to be triggered by individuals who suppress (i.e., network density – based on limited development of network ties and cultural homophily), have been shown by previous research to have further effects for the individuals involved: failing to reach out for social support to diverse others has been proven counterproductive for the advancement of minority groups in organizations (Ibarra, 1993). Homogenous support networks are beneficial for the advancement of white males, but less so for women and other minorities. Therefore, it is not unreasonable to link the cultural homophily effect we evidenced in our data with *lesser chances for promotion* and overcoming stereotyping and bias in organizational settings for minorities (Ibarra, 1993), as suppression increases the homogeneity of these minorities’ networks.

### Limitations and Directions for Future Research

Our study presents a number of limitations, and we tried to make a clear distinction throughout our discussion between what our data says and what it can imply – which could inform future research directions. We summarize these distinctions in the present section.

First, at the group level, our observed structural patterns point toward possible explanations why in diverse groups less information gets transmitted, and when it does, preferential information (which does not challenge fundamentally one’s pre-existing cognitive schemata) is easier transmitted. Also based on our results we can see that information has boundaries of transmission – everyone gets to keep their opinion, which possibly explains polarization of opinions in diverse groups. This translates into less use of diversity resources in superdiverse groups and lack of adaptation of minorities in moderately diverse groups. However, our design does not provide any information about the task-related networks, but focuses strictly on social support networks in professional groups. Furthermore, we did not collect any data on the actual content that flows in these networks, but focused only on the structural aspects of the networks. While our data does not directly measure the content transmitted, the structural effects that we evidenced have previously been shown to matter in group performance: in their 2014 paper, Tröster et al. demonstrated how the interplay between network structure and team composition influences the success of a diverse team.

At the individual level, we did not measure the evolution of well-being indicators, in order to be able to draw direct conclusions on how support seeking impacts then the well-being or adaptability of the individual. Furthermore, we measured habitual suppression only at the beginning of the study. While this is in general an accepted practice, Srivastava et al. (2009) showed there are changes in habitual suppression for college students between the first and fourth year of college. We considered that our time span is too short to justify such a change, and for reasons of time needed to fill the network rooster we did not measure it at two times. However, in line with research proposing that support networks are external emotion regulation strategies (Rimé, 2009), it would be interesting to observe in the future if there is a reciprocal effect between support networks and habitual suppression. In other words, could the size or diversity of one’s support network impact the use of habitual suppression? And how does this interplay between suppression and support networks influence one’s wellbeing, adaptation to a new group, or even position in the group? Longitudinal research in real groups could offer relevant insights on these questions.

Thirdly, building on the work of Tröster et al. (2014) and Stahl et al. (2010), we used Hofstede’s country values on the dimension individualism/collectivism (Hofstede et al., 2010) as a proxy for the value system in which someone got his/her primary socialization instead of measuring at individual level one’s declared level of individualism/collectivism. While this measurement choice does not come without limitations (e.g., subculture and individual differences within the same country), we opted for it as it indicated the background in which one was socialized and the implicit assumptions on self-expression vs. restraint embedded in that culture, which were relevant for the mechanisms we wanted to reveal. Furthermore, in using an existing indicator we limited collinearity effects in our design (i.e., the moderator was not measured through self-reports). Further research however can continue this investigation by comparing effects of individual-level measurements of individualism/collectivism with country-level, by extending the exploration to other cultural dimensions, as well as by looking into how relevant diversity faultlines (e.g., age, gender) impact the network behavior of actors (as we have only explored one type of diversity in our design, namely cultural). We acknowledge that since norms for emotion expression and suppression vary across genders – and these differences might be experienced differently in various age cohorts, as norms change over time, further explorations into these intersectional effects on support seeking behaviors are highly relevant for nowadays organizations and their diverse workforce.

Another interesting aspect that warrants interest in future research is the cultural context in which these multicultural groups operate, and whether that impacts the relationships under scrutiny. More to the point, our study was conducted in an individualistic cultural setting. It is not unreasonable to question whether the setting itself (through its salient individualistic values, which might contradict fundamental worldviews about relating practices held by participants coming from collectivistic cultures) impacted the support seeking behaviors of participants, for instance by enhancing the similarity effect of participants coming from collectivistic countries, or by increasing their perception of being outsiders. It would be interesting to explore in future research whether the effects we noticed in our study remain the same or follow different patterns if multicultural groups operate in a collectivistic cultural setting, as well as explore in-depth the psychological mechanisms at play behind these behaviors.

## Conclusion

We started our research from the assumption that if emotions convey meaning and are tools for social learning, they should help us in learning to navigate relationships with dissimilar others. However, if these dissimilar others do not share these emotions, it remains difficult to “ground” and establish close relations and provide support. In this respect, our findings indicate that people coming from individualistic cultures suffer a double-whammy: when suppressing, their members seek less support and tend to seek similar others for it when they do. Suppressing collectivists are less affected in absolute levels of connectedness, but still prefer culturally similar others as sources of support. In sum, we offer an emotion-based explanation to why people stick together with similar others in diverse groups and propose that learning to better cope with emotions can make us more open-minded toward diversity in professional settings.

## Data Availability Statement

The datasets generated for this study are available on request to the corresponding author.

## Ethics Statement

The studies involving human participants were reviewed and approved by the Vlerick Business School. The patients/participants provided their written informed consent to participate in this study.

## Author Contributions

LG was in charge of data collection and analysis. SB wrote the manuscript, with the two other authors reviewing it at the end. All three authors contributed to the research design.

## Conflict of Interest

The authors declare that the research was conducted in the absence of any commercial or financial relationships that could be construed as a potential conflict of interest.

## Footnotes

1 Because “culture consists of a commonly held body of beliefs and values that define the “shoulds” and the “oughts” of life” (Stahl et al., 2010, p. 691), cultural diversity increases divergent group processes (i.e., processes that bring different values and ideas into the team and juxtapose them with each other – Davison and Ekelund, 2004), such as creativity (positive) and conflict (negative). Systematic evidence (see Stahl et al., 2010 for a meta-analysis) points that multicultural groups experience more task conflicts than homogenous ones, but are also more creative. These processes, however, come at an emotional cost, as cultural diversity has a negative influence on the affective dimension of social integration (i.e., “the attraction to the group, satisfaction with other members of the group, and social interaction among group members” – O’Reilly et al., 1989, p. 22).

2 We built the moderately diverse-superdiverse dichotomy borrowing from the work of Vertovec (2007) in demographic, who defined superdiverse conditions as: “distinguished by a dynamic interplay of variables among an increased number of new, small and scattered, multiple-origin, transnationally connected, socio-economically differentiated and legally stratified immigrants” (Vertovec, 2007, p. 1024). The concept of super-diversity has been then borrowed by diversity research as a conceptual device with which to observe complex societies; it points to the changing conditions of diversity and the multiplication of variables, a phenomenon which differentiates today’s urban societies from previous demographic conditions (Vertovec, 2007).

3 When applying stochastic actor-based models it is crucial to check the convergence of the algorithm in order to see if the algorithm could be applied on the data or not. This check considers the deviations between simulated values of the statistics and their observed values. Ideally, these deviations should be 0. Because of the stochastic nature of the algorithm, when the process has properly converged the deviations are small but not exactly equal to 0. We calculated the averages and standard deviations of the deviations in RSIENA and combined these in a t-ratio (in this case, average divided by standard deviation). The overall maximum convergence ratio is the maximum value of the ratio average deviation standard deviation for any linear combination of the target values.
